# Inferring Users’ Social Roles with a Multi-Level Graph Neural Network Model

**DOI:** 10.3390/e23111453

**Published:** 2021-11-01

**Authors:** Chunrui Zhang, Shen Wang, Dechen Zhan, Mingyong Yin, Fang Lou

**Affiliations:** 1 Faculty of Computing, Harbin Institute of Technology, Harbin 150001, China; 16B903061@stu.hit.edu.cn (C.Z.); dechen@hit.edu.cn (D.Z.); 2 Institution of Computer Application, China Academy of Engineering Physics, Mianyang 621900, China; yinmy@caep.cn (M.Y.); louf108@caep.cn (F.L.)

**Keywords:** network representation learning, graph neural networks, social networks, social status and role inference

## Abstract

Users of social networks have a variety of social statuses and roles. For example, the users of Weibo include celebrities, government officials, and social organizations. At the same time, these users may be senior managers, middle managers, or workers in companies. Previous studies on this topic have mainly focused on using the categorical, textual and topological data of a social network to predict users’ social statuses and roles. However, this cannot fully reflect the overall characteristics of users’ social statuses and roles in a social network. In this paper, we consider what social network structures reflect users’ social statuses and roles since social networks are designed to connect people. Taking an Enron email dataset as an example, we analyzed a preprocessing mechanism used for social network datasets that can extract users’ dynamic behavior features. We further designed a novel social network representation learning algorithm in order to infer users’ social statuses and roles in social networks through the use of an attention and gate mechanism on users’ neighbors. The extensive experimental results gained from four publicly available datasets indicate that our solution achieves an average accuracy improvement of 2% compared with GraphSAGE-Mean, which is the best applicable inductive representation learning method.

## 1. Introduction

In recent years, online social networks have become more and more popular in enabling people to connect, communicate, and share information. At the same time, from the tremendous information in social networks, specifically that from the interaction between social network users, we can deduce the relations between users and the overall social structures. Furthermore, we can deduce a user’s social status and role, which reflect the position of an individual in society, as it may represent the honor or prestige corresponding to that social position. A user’s social role varies from one social networks to another. As a result, a person may behave differently in different networks, as the person’s role in a social network may have a different position compared to that of another network and thus its interaction with other roles in the corresponding network is distinct. For example, users of Weibo include celebrities, government officials, and social organizations. Meanwhile, social roles in a company include senior managers, middle managers, and workers. A celebrity in Weibo that is connected with numerous fans may be a worker in a company, where the person might be a worker that only connects to the boss. Studying users’ social statuses and roles is very helpful in order to gain insight into society as a whole as well as into managing social resources at the individual level. In particular, understanding users’ social roles is crucial to many social network applications such as targeted advertising and personalized recommendations.

Conventional approaches [1,2] propose applying data mining techniques to social network data in textual or categorical form to predict user attributes. However, in real social networks, the textual or categorical information does not usually reflect the user’s identity because it contains only partial information about users, as the relations between users are lose. In this paper, we analyzed the Enron emails dataset and extracted users’ features from their communication behavior. These features including neighbor influence, tie density, centrality, tie strength and trust, and connectivity, which correspond to a variety of key aspects of the social network. However, it is difficult to infer a user’s social role solely through the use of these original features. Hence, we need to find an effective feature-embedding method to help us infer the user’s social status and role.

In recent years, due to the graph structure’s powerful expression ability, it has been increasingly used in machine learning methods for graph analysis First of all, the graph neural network (GNN) is a widely adopted method devised for processing graph datasets for its high performance and interpretability. Secondly, Graph embedding is able to learn low-dimensional representations of graph nodes, while effectively preserving the graph structure. Recently, significant progress has been made in this emerging research area [3,4,5]. However, state-of-the-art methods still have obvious defects. For example, Kipf et al. [6] proposed a very popular variant of GNN named the graph convolutional network (GCN). Motivated by the first-order approximation of spectral graph convolutions, the authors introduced a simple layer-wise propagation rule. GraphSAGE [4] extended the GCN to the inductive learning task in large-scale graphs, which generates embeddings by sampling and aggregating features from a node’s direct neighbors. However, GraphSage uniformly aggregates each node’s neighbors which limits its ability to represent nodes’ features in social networks (e.g., email [7,8,9,10], webpage [11] and citation [12,13,14] networks). It does not take into account the fact that different neighbors’ nodes contribute differently to the representation of the given node. The graph attention network (GAT) [5] specifies different weights to different nodes in a neighborhood by leveraging masked self-attention layers to address the shortcomings of the GCN-based methods, which is applicable to both transductive and inductive problems. However, it cannot differentiate the importance of different levels of a given node’s neighbors. In social networks, the direct neighbors of counterpart entities are usually dissimilar due to the schema heterogeneity, AliNet [3] employs an attention mechanism to highlight k-hop distant neighbors and reduce noises. Their experimental results have shown its effectiveness on entity alignment across two knowledge graphs. Unfortunately, AliNet has to compute each hop neighbor, respectively, which increases its computational burden when the datasets become large. In order to effectively infer users’ social statuses and roles, we propose a novel inferring method that deals with the feature information of users by considering different contributions of a given node’s neighbors. We propose adopting the attention mechanism to emphasize the difference of the aggregation functions at each layer. Then, we use the gate mechanism to learn the weight of the multi-level expressions to generate the final network embedding.

The contributions of this paper are summarized as follows:

1. The use of behavior-based local features and social principle-based global features is proposed to improve the feature expression ability. We used the Enron email dataset as an example to show how these features affect the feature expression ability.
2. We designed a novel network embedding learning model for inferring users’ social statuses and roles which takes users’ local and global features and relations as inputs. In our model, considering the directed relations and large volume of edges and nodes in the email social networks, we used spatial convolution GCN to deal with large-scale social networks representation learning tasks. We emphasized the different contributions of neighbors to the central node by involving the attention mechanism and gate mechanism. Our model achieves a better user representation learning ability through the use of an attention and gate mechanism.
3. Extensive experiments were carried out to show the effectiveness of our solution. The comparative results demonstrate the superiority and robustness of our model.

The rest of this paper is organized as follows. We review the related works in Section 2. Section 3 explains the preliminaries. In Section 4, we describe our solution and implementations in detail. The experimental evaluations are shown in Section 5. Finally, Section 6 concludes this paper.

## 2. Related Work

In research on social networks, the problem of users’ social role identification is highly significant in predicting the behavior of users and inferring the relationship between them. There have been efforts to study social network role identification. Most of these approaches can be grouped into two categories: (1) identifying users’ roles according to the analysis of social network structure or users’ social position—namely, the method of statistical analysis; and (2) using machine learning methods to identify users’ roles.

Several previous works have been carried out to study the effects and patterns corresponding to a variety of key aspects of social networks, including neighborhood influence, tie density, etc. For example, Zhu et al. [15] used Pangerank centrality to distinguish nodes across communities. Aliabadi et al. [8] classified professional roles using node degrees, cluster coefficients, betweeness, HITS, and PageRank. In [16], local structural information and network influence is represented by a probabilistic model in order to infer unknown social statuses and roles of users. All these works assume that the homophily pattern indicates the similarity of the professional roles among users. These works rely on specific pre-defined network patterns. Other works use attributes such as textual content or the topics of the links in social networks (e.g., the RART [1]). However, this type of modeling necessitates the collection of textual features of social network data (e.g., email content), which becomes more and more difficult due to increasing public privacy concerns in the real world.

The *Struc2Vec* [17] model establishes a hierarchical similarity measurement. It can capture the structural node similarity by considering a hierarchical metric defined by the degree of ordering of a sequence of nodes. Jin et al. [18] proposed the use of EMBER for large-scale email communication networks. This model can create an email-centric in/out degree histogram of nodes in the network and automatically capture behavioral similarity, allowing it to distinguish employees with different hierarchical roles. Unfortunately, experiments show that the in/out degree histogram of nodes is biased, making it necessary to use a pre-defined balance coefficient according to the given dataset to fine-tune the classification results. This means that EMBER is not adaptable to alternative datasets. Other works, such as LINE [19], DeepWalk [20], and *Node2Vec* [21], consider the similarity of node proximity. Experiments show that they perform worse than Ember when they are applied in a user professional role inference scenario. The above methods assume that there is a homophily pattern to users’ social roles in a social network. However, the pattern is weak and thus it is not possible to independently infer users’ professional roles effectively.

Graph neural networks (GNNs) have shown outstanding performance in representing nodes. Velickovic et al. [5] proposed the use of GAT on the basis of GCN. GAT uses an attention mechanism to emphasize nodes that have a greater impact on entities to obtain representations. Xu et al. [22] proposed the use of graph wavelet neural network (GWNN) which replaces the graph Fourier transform with a graph wavelet transform for analyzing a graph network. Sun et al. [3] proposed AliNet, which combines the attention mechanism with a gating mechanism to generate node representation, which is used to align a knowledge graph. However, AliNet’s inputs are two graph data. Although the model can be modified to generate a single graph node representation, it will cause a large computational overhead preventing its application to large-scale social networks. In addition, social networks can be dynamic. For newly added nodes, AliNet needs to retrain the entire network to obtain representations, which incurs high computation overhead. William et al. [4] proposed GraphSAGE, which learns a function that samples and aggregates features from a node’s local neighborhoods to generate embedded features. In addition, it can efficiently generate embeddings for first-seen nodes. Hence, GraphSAGE supports large-scale dynamic social networks. However, it ignores the influence of different neighbor nodes on the entity when aggregating features from a node’s direct neighborhoods. Having reviewed the aforementioned methods, we propose the use of GraphSAGE as a basic model to train a function that generates node embeddings. Meanwhile, we integrate the attention and gate mechanisms to learn node representations, emphasizing the importance of neighborhoods that have a greater impact on the node.

## 3. Preliminary

To ease the understanding of mathematical derivation in this paper, we summarize the notations used in Table 1.

### 3.1. Sociology Theories

#### 3.1.1. Triadic Closure

Triadic closure follows the most basic rules in social network theory, which indicates the nodes’ latent social relationships [16]. It has been widely used to analyze social ties. The basic pattern of triadic closure in social networks can be quantitatively measured by **the Local Clustering Coefficient**(**LCC**) [23,24] which is computed as

(1) $$LCC_{i}=\frac{2\cdot |\left\{e_{j,k}:j,k\in N_{v_{i}}\right\}|}{\left|N_{v_{i}}\right|\cdot \left(\left|N_{v_{i}}\right|-1\right)}$$

where $N_{v_{i}}$ is the set of a given node $v_{i}$’s neighbors; $e_{j,k}$ is the edge connecting nodes *j* and *k*; and *j* and *k* are neighbors of *i*.

$LCC_{i}$ is in the range of [0,1] which measures the closeness of neighbors to a clique in a social network. Intuitively, this indicates the number of triangles connected to node $v_{i}$.

#### 3.1.2. Reach

**Degree centrality** (**DC**) [25] is defined as the number of neighbors for a given node *v*, which is used to measure the reach of node *v* in a social network:

(2) $$DC_{v}=\left|u,\forall u\in N\left(vs.\right)\right|,\forall vs.\in V$$

where $\left|\cdot \right|$ is the size of the neighbor set, i.e., the number of neighbors of node *v*.

Previous works have shown that the distribution of **DC** can be used to classify different users’ social roles. Zhao et al. [16] reported that the distribution of the **DC** of the *Research & development* role has a much steeper shape, with 80% of users having node degrees inferior to 200.

#### 3.1.3. Tie Strength and Trust

**Embeddedness** (called **Emb** for short) can be used to measure the tie strength and trust of a given node $v_{i}$ which is defined as [16]

(3) $$Emb_{v_{i}}=\frac{1}{\left|N_{v_{i}}\right|}\sum_{v_{j}\in N_{v_{i}}}\frac{\left|N_{v_{i}}\cap N_{v_{j}}\right|}{\left|N_{v_{i}}\cup N_{v_{j}}\right|}$$

**Embeddedness** score shows the degree to which individuals are enmeshed in social networks [26] which can also represent a trust relationship between any pair of nodes in a social network.

#### 3.1.4. Structural Holes

**Figure 1:**
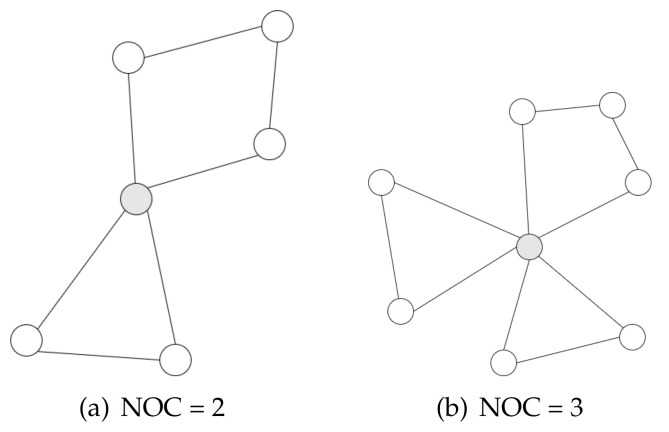
Networks with NOC = 2 and NOC = 3 of gray nodes.

In sociology, a structural hole represents a user who connects with multiple non-interacting parties. The name comes from the notion that an ’empty space’ will be left in the network if such a user is removed. Typically, the **Number of Communities (NOC)** is used to represent the property of structural holes [16,27]. A node with a high NOC indicates that it is structurally important because it connect diverse regions in the social network. As shown in Figure 1, two gray nodes with **NOC = 2** (Figure 1a) and **NOC = 3** (Figure 1b). Obviously, computing the NOC of a node involves the detection of communications in a given social network. Previous works [27,28] have shown it is a highly consuming computation process. On the other hand, NOC alone has many limitations to represent the property of structural holes in a large-scale social network, thus it is hardly applicable in real-world applications. We refer readers to [28] for more details about social principles and theories.

### 3.2. GNN

Although traditional deep learning methods have achieved great success in extracting the characteristics of Euclidean spatial data, performance in the processing of non-Euclidean spatial data is still not satisfactory. GNNs can successfully solve this problem; their application can aid in research on pattern recognition and data mining. We mainly discussed two kinds of graph neural networks that are relevant to this paper: GCN and GAT.

#### 3.2.1. GCN

The core idea of graph convolutional networks is to learn a function f(.) through which the node *v* in the graph can aggregate its own feature $x_{v}$ and its neighbor feature $x_{u}$ (u∈N(v)) to generate a new representation of the node *v*. GCNs can be divided into two categories: spectrum-based and space-based methods. A common disadvantage of spectral-based graph convolutional neural network methods is that they require the entire graph to be loaded into memory to perform graph convolution, which is inefficient when dealing with large graphs. The idea of space-based graph convolutional neural networks is mainly motivated by traditional convolutional neural networks operated on images. The difference is that the space-based graph convolutional neural network defines graph convolution based on the spatial relations of nodes. For a generic graph, the spatial-based graph convolution aggregates the central node representation and adjacent node representations to obtain a new representation of the node.

Neural Network for Graphs (NN4G) [29] was the first study to research spatial-based convolutional GNNs. After NN4G, a number of approaches [4,30,31,32,33] have been proposed. Among them, GraphSAGE is a typical method that mainly updates nodes’ representations by sampling and aggregating their neighbors’ representations. The first candidate aggregator function takes the element-wise mean of the vectors in $\left\{h_{u}^{k-1},\forall u\in N\left(v\right)\right\}$, computed by

(4) $$\begin{array}{c} h_{v}^{l}\leftarrow \sigma \left(W\cdot \mathrm{MEAN}\left(\left\{h_{v}^{l-1}\right\}\cup \left\{h_{u}^{l-1},\forall u\in N\left(v\right)\right\}\right)\right. \end{array}$$

where σ(·) is an activation function, $h_{v}^{l-1}$ is node *v*’s previous layer representation, and N(v) is the sampling neighbor set of node *v*.

#### 3.2.2. GAT

Graphic attention network (GAT) uses an attention mechanism to determine the weight of nodes’ neighbors when aggregating feature information. It has the advantage of being able to amplify the impact of the most important parts of the data. The graph convolution operation of GAT is defined as

(5) $$\begin{array}{c} h_{v}^{l}=\sigma \left(\sum_{u\in N(v)}\alpha_{vu}^{\left(l\right)}z_{u}^{\left(l-1\right)}\right) \end{array}$$

where σ(·) is an activation function, N(v) represents the set of neighbors of the given node *v*, $z_{u}^{\left(l-1\right)}$ is a linear transformation of (l−1)-th layer nodes embedding $h_{u}^{l-1}$ and $\alpha_{vu}^{\left(l\right)}$ is the attention score that is calculated by an attention function that adaptively controls the contribution of the neighboring node *u* to node *v*. The way in which we calculate attention weights $\alpha_{vu}^{\left(l\right)}$ is described in Section 4.

## 4. Our Proposed Solution

In a social network, our goal is to infer each node’s social status and role. Our method consists of two steps: feature extraction and role inference.

### 4.1. Feature Extraction

**Figure 2:**
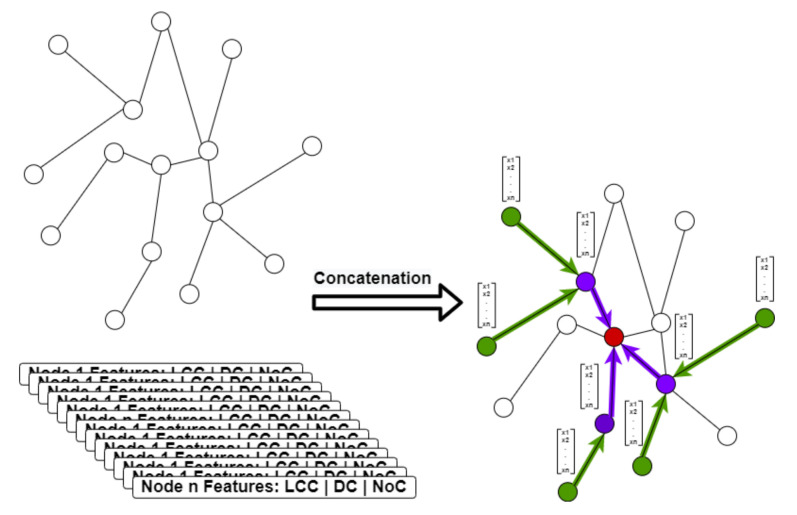
The overview of the node feature extraction.

We describe each node with a feature vector. We analyze the user’s behavior characteristics according to the specific network and downstream tasks (e.g., user’s social role inference). Furthermore, we extract features which contain LCC, DC, Emb, and NOC according to the sociology theories described in Section 3. In order to improve the expression ability of our eigenvector, we concatenate these features to form a fixed-sized vector for each node. Figure 2 shows the main steps of the node’s feature extraction and concatenation.

### 4.2. Role Inference

**Figure 3:**
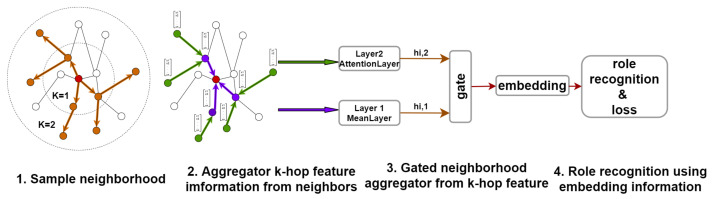
The overview of our role inference network architecture.

The key idea of this step is to aggregate feature information from a node’s local neighborhood while highlighting the importance of each neighbor. In our solution, the nodes’ embeddings are generated by controlling the aggregation of both direct and multi-level distant neighborhood features using a gating mechanism. The role inference network architecture is illustrated in Figure 3.

#### 4.2.1. Neighborhood Sampling

Because the number of neighbors of each node may be very large and variable in a given large graph, sampling the neighbors of each node will cause the input size to be fixed and make computation easier. This neighborhood is conceptually similar to the receiving field [34] in the convolutional neural network, whose size is fixed in each layer of a network.

Specifically, considering users’ sending or receiving emails behavior in email social networks, we first divided the neighbors of each node into in-degree neighbors and out-degree neighbors. We defined N(v)+ ( N(v)−) as a fixed-size uniform draw from the set {u∈V,(u,v)∈E+} ({u∈V,(u,v)∈E−}), respectively, where E− denotes the set of incoming edges (e.g., receiving emails) in the social network graph (G), while E+ denotes the set of outgoing edges. The size of N(v)+ (N(v)−) is set to *S*. Two conditions exist: (1) when the number of neighbors is less than *S*, we adopt the uniform sampling method with placement until *S* neighbors are sampled; and (2) when the number of neighbors is greater than *S*, uniform downsampling is used. This neighborhood sampling allows us to store neighborhood information as dense adjacency lists, which drastically improves computational efficiency.

After constructing the neighborhood for each target node using the above sampling method, the features of the nodes in the neighborhood are aggregated and transmitted to the graph neural network layer. By using the hierarchical propagation mechanism of neural networks, each target node iteratively aggregates the features further and further away in the graph, thus obtaining the node representation at each layer in turn.

#### 4.2.2. One-Hop Neighborhood Aggregation

The input of our model is a digraph G(V,E) and the initial feature matrix $H^{0}$, which is the node embedding of the first layer of the graph neural network. In order to aggregate the features of the one-hop neighborhood, we introduced a GNN layer, denoted by *Meanlayer*. It is worth noting that previous works iteratively update a node’s representation by aggregating information between the node and its neighbors without taking into account how different it is from its neighbors. *MeanLayer* overcomes this shortcoming by only aggregating the features of the surrounding neighbors, thus ensuring that the iteratively updated node information includes only first-level neighbor features. In contrast to Equation (4), the hidden representation of *MeanLayer* is computed by

(6) $$\begin{array}{c} h_{N(v)}^{1}\leftarrow Mean\left\{h_{u}^{0},\forall u\in N\left(v\right)\right\} \end{array}$$

#### 4.2.3. Attention for Distant Neighborhood

There is an exponential relationship between the number of neighbors further away from the node and the number of first-level neighbors. We iteratively update the embedding representation of each node and obtain the high-level representation of the central node by aggregating its first-level neighbors’ features. As shown in Figure 3, it is worth noting that, after the *Meanlayer*, the first-level neighbors’ representations ($h_{N(v)}^{0}$) actually contain the features of the second-level neighbors ($h_{N(v)}^{1}$) of the central node. However, it is obvious that the distant neighbors contribute differently to the central node, so we use the attention mechanism to emphasize these useful neighbors. The attention function in Equation (5) does not take into account the difference between the central node and its neighborhood because it applies a shared linear transformation to each node. Hence, we use one matrix for the linear transformation of the center node and another one for those of its neighbors to calculate the original attention score $e_{ij}^{\left(l\right)}$ at the *l*-th layer. This layer is denoted by AttentionLayer. The AttentionLayer typically performs two calculations: (1) that of the original attention score between any two pair of nodes; and (2) that of the final attention score of any pair of nodes. The original attention score is computed by

(7) $$\begin{array}{c} e_{ij}^{\left(l\right)}=\mathrm{LeakyReLU}\left({\vec{a}}^{{\left(l\right)}^{T}}\left(W_{1}^{\left(l\right)}h_{i}^{\left(l\right)}∥W_{2}^{\left(l\right)}h_{i}^{\left(l\right)}\right)\right) \end{array}$$

Equation (7) first concatenates the linear transformation of the embeddings of the pair. Here, ∥ stands for concatenating. Then, we take the dot product of the new embedding and a learnable weight vector ${\vec{a}}^{\left(l\right)}$, which is to generate the feature vector of each node’s attentions. Finally, a *LeakyRelu* activation function is applied to deal with the non-linearity problem of the inputs. We used the Softmax function to compute the final attention score $\alpha_{ij}^{\left(l\right)}$ between nodes *i* and *j* at the *l*-th layer, which is computed by

(8) $$\begin{array}{c} \alpha_{ij}^{\left(l\right)}=\frac{\exp\left(e_{ij}^{\left(l\right)}\right)}{\sum_{k\in N(i)}\exp\left(e_{ik}^{\left(l\right)}\right)} \end{array}$$

Note that the Softmax function is a function that turns a vector of *K* real values into a vector of *K* real values that sum up to 1. The input values can be positive, negative, zero, or greater than one, but the softmax transforms them into values between 0 and 1, so that they can be interpreted as probabilities.

#### 4.2.4. Multi-Hop Neighborhood Aggregation Based on Gate Mechanism

The final representation of a node should contain information about all its neighbors at different levels. We propose using the gate mechanism to control the different contributions of the neighbors at different layers to the node. The gate mechanism takes different levels’ feature vectors as inputs, and learns a weight matrix to control the output. Taking two levels as an example, the gate function is:

(9) $$\begin{array}{c} z_{v}=g\left(h_{v}^{2}\right)\cdot h_{v}^{1}+\left(1-g\left(h_{v}^{2}\right)\right)\cdot h_{v}^{2} \end{array}$$

where $g\left(h_{v}^{2}\right)=\sigma \left(M\cdot h_{v}^{2}+b\right)$ is the gate used to control the combination of one-hop and two-hop neighborhoods, where M is the weight matrix, and *b* is the bias vector, and σ is an activation function. In our model, we use LeakyReLU as the activation function. This layer is denoted by GateLayer.

Algorithm 1 describes the embedding generation process using forward propagation. We take the graph, G=(V,E), and all the nodes’ features, $x_{v}$, ∀vs.∈V, as inputs. We assume that the model has already been trained and that the parameters are fixed. Each step in the outer loop of Algorithm 1 proceeds as follows: First, we use the neighbor sampling strategy in Section to uniformly sample two fixed-size sets from the in-degree and out-degree neighbors, instead of using full neighborhood sets. Then, for each node v∈V, we aggregate the representations of its neighborhoods, $\left\{h_{u}^{k-1},\forall u\in \left(N\left(v\right)+,N\left(v\right)-\right)\right\}$, into a vector $h_{N(v)}^{k}=\left[h_{N(v)}^{k}+,h_{N(v)}^{k}-\right]$. We use the Mean aggregation strategy when k=0 (step 9). In other cases, we adopt the Attention aggregation strategy (step 10). Note that the *k*-th aggregation depends on the k−1-th generated representations. For the first layers (k=0), we use the input node features as the node representations. After the aggregation step, we applied a linear transformation with a nonlinear activation function σ to the aggregated neighborhood vector $h_{N(v)}^{k}$ (steps 11 and 12), which will be used in the next step of the algorithm (i.e., $h_{u}^{k-1},\forall u\in N\left(v\right)$). Finally, the gate function is used to control all representations $\left\{h_{v}^{1},\ldots ,h_{v}^{K}\right\}$ so as to obtain node *v*’s final representation $z_{v}$ (step 15).
**Algorithm 1** Our embedding generation algorithm for directed graphs.**Input:**Digraph G(V,E); hop *K*; input features $\left\{x_{v},\forall vs.\in V\right\};$ weight matrices $W^{k},\forall k\in \left\{1,\cdots ,K\right\}$; non-linearity σ; the aggregator functions: *MeanLayer* and *AttentionLayer*; the gate function: *gate*; the concatenate function: *Concat*; the neighborhood sampling function $N:vs.\to 2^{\nu }$; the weight coefficient: μ**Output:**      Node representations $z_{v}$ for all v∈V
1
$\;\;h_{v}^{0}\leftarrow x_{v},\forall vs.\in V$ 2FOR
k=1…K DO 3FOR
v∈V DO 4IF
k==1 DO
5
Aggregator=MeanLayer; 8ELSE DO
5
Aggregator=AttentionLayer;
9
$\;\;\;\;h_{N(v)}^{k}+\leftarrow Aggregator\left(\left\{h_{u}^{k-1},\forall u\in N\left(v\right)+\right\}\right);$
10
$\;\;h_{N(v)}^{k}-\leftarrow Aggregator\left(\left\{h_{u}^{k-1},\forall u\in N\left(v\right)-\right\}\right);$
11
$\;\;h_{N(v)}^{k}\leftarrow Concat\left(h_{v}^{k-1}+,h_{v}^{k-1}-\right);$
12
$\;\;h_{v}^{k}\leftarrow \sigma \left(W^{k}\cdot h_{N(v)}^{k}\right)$ 13   END
14   
$h_{v}^{k}\leftarrow h_{v}^{k}/{∥h_{v}^{k}∥}_{2},\forall vs.\in V$ 15 END
16
$z_{v}\leftarrow gate\left\{h_{v}^{1},\ldots ,h_{v}^{K}\right\},\forall vs.\in V$


#### 4.2.5. Learning the Parameters

The output representations, $z_{u}$, ∀vs.∈V are computed with a graph-based loss function. The parameters (e.g., ${\vec{a}}^{\left(k\right)}\forall k\in 1,\cdots ,K$) and the weight matrices ($W^{k}$, ∀k∈1,⋯,K) are tuned via the stochastic gradient descent method:

(10) $$\begin{array}{cc} J_{G}\left(z_{u}\right)= & -\log\left(\sigma \left(z_{u}^{⊤}z_{v}\right)\right)-\\ \; & Q\cdot E_{v_{n}∼P_{n}\left(v\right)}\log\left(\sigma \left(-z_{u}^{⊤}z_{v_{n}}\right)\right) \end{array}$$

where *v* is a node that can reach *u* with a fixed-distance random walk, σ is an activation function (e.g., LeakyReLU), $P_{n}$ is a negative sampling probability, and *Q* is the number of negative samples. We can replace the loss function (Equation (10)) with other forms (e.g., cross-entropy loss) on a specific downstream task to make the representations suitable for task-specific objectives.

## 5. Experimental Evaluations

In this section, we first analyze the feature extraction procedure for the Enron email dataset. Then, we describe the experiments performing role inference tasks.

### 5.1. Feature Extraction on Enron

#### 5.1.1. Enron Data Preprocessing

Email is an important means of information exchange meaning that a dataset of emails can be representative of a social network. The Enron dataset is the mail web logs of Enron personnel, where more than 500 thousands emails communicated between 151 users are collected. We remove files with irregular or empty email addresses. In the remaining files, the suffix “@enron.com” mailbox is treated as internal staff email and only records that have at least one mailbox suffix “@enron.com” of the sender and addressee were analyzed. We define a user as a node, and the mail sent between users is defined as a directed edge-connecting two nodes. Thus, the entire communication network can be constructed. Of course, if both parties to the communication are internal employees of the company, we can also abstract the internal communication network from it. Then, we can extract the data we need from the corresponding network.

#### 5.1.2. Users’ Social Role Levels

When we perform the role inference task in social networks, the position of each user is different and it is unrealistic to infer the role and position in detail. Therefore, users need to be roughly divided into several levels. For the Enron dataset, we standardized them and divided professional roles into three levels based on the existing literature [35]. These levels are senior managers, middle managers and workers. These divisions can enable us to clearly classify employees and facilitate the inference of role identities. We match each professional role with a set of keywords to divide users into different levels. However, due to the complexity of the names of professional roles in real scenarios, it is necessary to manually verify the classification results.

#### 5.1.3. Feature Selection

We take into account the privacy protection of users, so we avoid using any textual information about users and shift our attention to the structural features of the user’s communication network. As for email networks, we can extract some features from internal communication networks or external communication networks. These include the internal clustering factor, in-degree, out-degree, number of CC emails, and number of internal contacts. However, since the amount of information contained in the Enron dataset is relatively small and incomplete, we only extracted 46 available features. There may be some interdependence between these features. In order to make the features more comprehensively representative of the user, we filtered out dependent features according to their correlation coefficients. The 13 features we selected are shown in Table 2.

**Figure 4:**
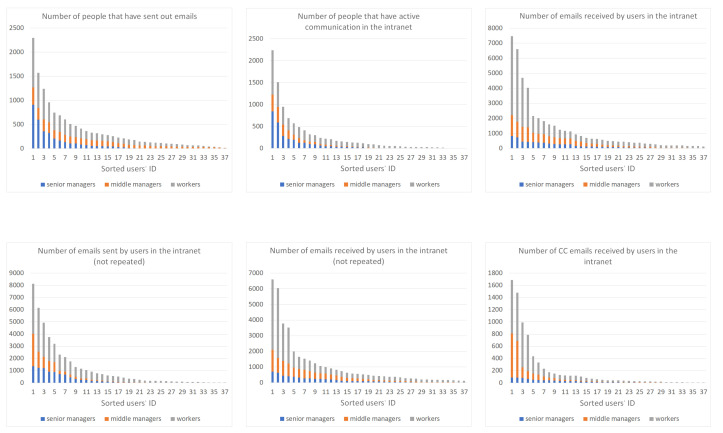
The features of different levels.

We selected six features, as shown in Figure 4. These features are related to the number of emails sent or received, the number of people actively communicating, and the number of CC emails sent or received. We can clearly observe that people of different levels show obvious differences in their features. For example, it is evident that users on the worker level usually receive many emails compared to those on the other levels, while senior managers rarely receive CC emails. This further shows that the features we selected are meaningful when completing role inference tasks. Moreover, previous work [16,28] also analyzed the major social principles and theories that are closely related to user social roles. From their analysis, various patterns were found to be represented in different social properties, and thus can be utilized to classify social roles. Based on this, we combined one of the sociological features ( LCC ) with our structural features to improve the expression ability of the user’s feature.

### 5.2. Datasets Description

In addition to the Enron email dataset, we used another three publicly available social network datasets to further evaluate our method.

The details of these datasets are described as follows, and the statistics are summarized in Table 3.

- **Cora** is a citation network where nodes are research papers on different topics and directed edges are citations between papers. Each paper is represented by a 1433-dimensional word vector that we use to represent the attributes of the node. The subjects are used as the labels of the nodes.
- **Citeseer** is also a citation network. The contents of the papers are used as node attributes and the topics are used as class labels. In this network, nodes represent research papers, and the directed edges are citations between papers.
- **WEBkb** contains webpages from four universities where each webpage was treated as a node with a unique string identity. Web pages are also labeled as professor, student, project, or other pages with binary features indicating whether each word in a vocabulary is present or not. The directed edges indicate linking (jumping) relations between the webpages.

The proposed solution was compared with seven related methods, which are exemplified as follows:

- **Ember** [18] captures employees’ behavioral similarity and produces embeddings which distinguish different hierarchical roles.
- **Strcut2vec** [17] constructs a multi-layer graph with hierarchical node similarity measurement to encode structural similarities into node features.
- **SDNE** [36] uses a semi-supervised deep model to capture highly non-linear network structures.
- **GWNN** [22] is a novel graph convolutional neural network (CNN) leverages graph wavelet transform.
- **GCN** [6] is the variant of CNN on images, but directly computes features on graphs and encodes both the local graph structure and the features of nodes.
- **GAT** [5]: GAT can specify a different weight for neighboring nodes and leveraging masked self-attentional layers to address the shortcomings of graph convolutions-based methods.
- **GraphSAGE-Mean** [4]: GraphSAGE iteratively generates embedded nodes by sampling and aggregating the local neighborhood features of nodes. Then, it learns the node embedding by maintaining network structure information. GraphSage has a variety of aggregation functions. The mean aggregation function is used in the experiments, and hence, we call this model GraphSAGE-Mean.

To ensure fair comparison, we used the optimal default parameters of the existing methods. As EMBER is a proposed embedding method for the mail domain, we only conducted experiments on the Enron mail dataset.

### 5.3. Experiment Settings

Our goal was to compare the accuracy of our method to that of others in a node classification task. The above node embedding methods can be formally divided into two categories: (1) node embedding based on GNN (including GCN, GAT, GWNN, GraphSAGE-Mean, AliNet-Cla, and our own method), using the results of Softmax as the basis for classification; and (2) a method based on network structure, including SDNE, EMBER and Struct2vec. In this method, each node will be encoded as a low-dimensional vector. We then added a classifier to make it suitable for a node classification task. Here, we chose SVM to be the classifier model.

Concerning dataset partitioning, we partitioned each dataset into a training set, verification set and test set. To achieve high training efficiency, we computed a threshold value *x* and selected *x* nodes for each category as the training set. The proportion of the training set was approximately 0.7. For the remaining nodes, we divided them into the verification set and the test set in a ratio of 1:2.

In the experiment, we set the hop K = 2 and the number of sampling neighbors as S1 = 10 and S2 = 25. The dimension of the hidden layers was the number of label classes.

Here, we report the accuracy (F1-score) of the use of the datasets for assessing users’ role inference performance. Higher accuracy indicates a better performance. We present the results of our experiments in Table 4. The best performance scores are reported in bold characters. For node classification tasks, our approach performs well on all benchmark datasets. Compared with GraphSAGE-Mean, which has the best average accuracy compared to the baseline methods, our method still shows an average accuracy improvement of 0.02. In particular, on the Enron dataset, compared with GraphSAGE-Mean, our method achieves a 10% gain. Compared with GCN, it achieves a gain of 0.05. On the Cornell dataset, compared with GCN, our method achieves an accuracy improvement of 0.2. These data already demonstrate the superiority of our approach. For the Citeseer dataset, due to the overlap of classification when collecting paper citation relationships, this dataset is less general and all methods achieve poor accuracy. On the Cora, Wisconsin, and Cornell datasets, our method shows the best accuracy, because we fully consider the relationship between neighbors of the same level and the relationships among different levels. These results further demonstrate the effectiveness of our use of attentional mechanisms as well as gate mechanisms. Several structure-based embedding methods (such as Struct2Vec and SDNE) are not effective on these datasets because they only consider the local relations of nodes. The average accuracy of GAT is less than that of GCN, which indicates that the nodes differ less from their immediate neighbors than from their distant neighbors, meaning that it may not be necessary to select the relevant neighbors based on their attention mechanism. This is why we chose to use the GCN layer instead of the GAT layer for the one-hop neighborhood aggregation. Compared with GCN, which iteratively aggregates neighbors’ features, our method shows an average accuracy improvement of approximately 0.04. These results further support our idea: for neighbors of the central node, we first considered aggregating the features among neighbors of the same level, then considered integrating the aggregation of features of different levels.

### 5.4. Analysis

#### 5.4.1. Aggregation Strategies of Multi-Hop Neighborhood

To gain a deeper insight into our model, we designed three variants based on our method and using different strategies for aggregating multi-hop neighborhoods. The first method, denoted by *OurMethod-Mean*, applies MeanLayer in each level, followed by a GateLayer. The second method, denoted by *OurMethod-Att*, replaces MeanLayer with AttentionLayer to aggregate the features of one-hop neighbors. The third method, denoted by *GraphSAGE-Mean*, applies meanaggregator [4] to iteratively aggregate the information of each hop neighbor without using the gate mechanism (its aggregation representation contains the information of all previous neighbors). The results of our experiment are shown in Table 5. It can be seen that *OurMethod-Mean* does not perform better, indicating that the performance of the use of MeanLayer to aggregate higher-level neighbor information is poor, as not every higher-level neighbor contributes to the representation of the central node. The accuracy of *OurMethod-Att* is slightly lower than that of our method. This result shows that it may not be necessary to select the related neighboring nodes based on an attentional aggregation strategy because the nodes are less different from their immediate neighbors. For a similar reason, the accuracy of *GraphSAGE-Mean* is also slightly lower than that of our method. Based on these results, we conclude that the multi-level neighbor features indeed contribute to the efficient embedding representation of nodes, and that the gate and attention mechanisms can better capture the important information of distant neighbors than the basic idea of *GraphSAGE-Mean*.

#### 5.4.2. Impact of the Number of Layers K

**Figure 5:**
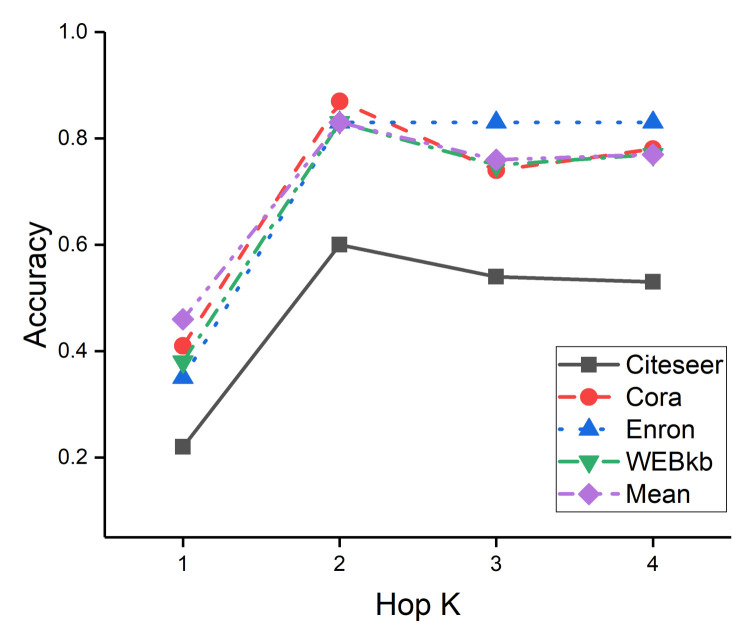
Accuracy on the baseline dataset under different K.

In our method, the result of the k-th layer denotes the aggregation information of the k-hop neighbors. Figure 5 shows the accuracy of our method with 1–4 layers on the baseline dataset. Our method with two layers achieves the best performance across all the datasets. We observe that the proposed method’s performance declines with the increasing of layers. Although deeper layers allow us to capture distant neighborhood information by layer-to-layer propagation, the use of such distant neighbors would introduce a large amount of noise. Therefore, aggregating two-hop neighborhood information is sufficient for node embedding.

#### 5.4.3. Performance Based on Different Layers

In our method, meanlayer was used to aggregate the information of one-hop neighbors, while attentionlayer was used to aggregate the information of k-hop neighbors. Attentionlayer can be also used to aggregate the information of one-hop neighbors. Here, we compare the time consumption of the first layer under meanlayer and attentionlayer. On the Cora dataset, the meanlayer takes approximately 0.6 s. Surprisingly, attentionlayer takes approximately 0.21 s. However, meanlayer and attentionlayer have the same time complexity of $O(|V|FF^{{}^{\prime }}+|E|F\prime )$. It can be observed that the time consumption of attentionlayer is tens of times that of meanlayer. This is because attentionlayer introduces one more matrix, *W*, which represents the attention scores of different neighbors when calculating the first layer’s embedding. The single meanlayer consumes |V|·F·F′ multiplication operations and |E|·F′ addition operations. Matrix *W* causes attentionlayer to produce an additional multiplication operation of |V|·F·F+2·|V|·F·S. When the dimension of the hidden layer’s output embedding is low and the dimension of the hidden layer’s input eigenvector is too large, the time cost factor between attentionlayer and meanlayer, denoted by $\frac{|V|\cdot F\cdot F+2\cdot |V|\cdot F\cdot S}{\left|V\right|\cdot F\cdot F\prime }$, will be very large. Furthermore, when the factor is too large, the computation of the weight matrix will create an unsustainable time cost, which is another reason why we chose to use meanlayer rather than attentionlayer for single-hop neighborhood aggregation.

Table 6 shows the time consumption of each layer on four benchmark datasets. We can observe that there is little difference in time between the MeanLayer and the Attentionlayer, as the MeanLayer transformed high-dimensional data into low-dimensional data, which allow us to use the attention mechanism for the aggregation of multi-jump neighbors. As for the GateLayer, since it only performs simple linear calculations, its time consumption is very small. Therefore, for a graph network containing tens of thousands of nodes, the length of time of an epoch in our method can be controlled to within an acceptable time range.

## 6. Conclusions

In this paper, we propose the use of behavior-based local characteristics and social principle-based global characteristics to improve features’ expression ability. Taking an Enron email dataset as an example, we analyze a preprocess mechanism used for social network datasets that can extract users’ dynamic behavior features. The analysis results indicate that generating one fixed-size feature vector for each node by concatenating different levels of user features is effective. Furthermore, we propose the use of a novel network model that integrates attention and gate mechanisms for inferring users’ roles. This is able to better learn network representations on large-scale graphs with an inductive approach. Experimental evaluations show that our solution can achieve a 2% improvement in accuracy compared with GraphSAGE-Mean, which is the best applicable inductive representation learning method. In future work, we plan to extend our model to deal with dynamic email network datasets.

## Figures and Tables

**Table 1:** Summary of notations.

Notations	Description
G	Graph network
V, E	The set of nodes and edges, resp.
|V|, |E|	The number of nodes and edges, resp.
N(v)	The neighbor set of node *v*
*x*	Feature matrix
*F*	The dimension of the GNN layer input eigenvector
$F^{{}^{\prime }}$	The dimension of the GNN layer output embedding
*S*	The number of sample neighbors
N(v)+	The in-degree neighbor set of node *v*
N(v)−	The out-degree neighbor set of node *v*
$h_{v}+$	The in-degree embedding of node *v*
$h_{v}-$	The out-degree embedding of node *v*
μ	The weighting factor between in-degree and out-degree embedding
$h_{v}$	The node *v*’s hidden layer output embedding

**Table 2:** The features of the users.

ID	Feature
1	Number of people that have sent out emails
2	Number of people that have received emails from other users
3	Number of people that have active communication in the intranet
4	Number of people actively communicating with users in the intranet
5	Number of emails received by users in the intranet
6	Number of emails sent by users in the intranet (not repeated)
7	Number of emails received by users in the intranet (not repeated)
8	Number of CC emails sent by users in the intranet
9	Number of CC emails received by users in the intranet
10	Number of people that have active communication in the external network
11	Number of people actively communicating with users in the external network
12	Number of CC emails sent by users in the external network
13	Number of CC emails received by users in the external network

**Table 3:** Descriptions of benchmark datasets.

Datasets		Nodes	Edges	Attributes	Labels	Field
Enron		125	1349	13	3	Email
Cora		2708	5429	1433	7	Citation
Citeseer		3312	4732	3703	6	Citation
WEBkb	Cornell	195	304	1703	5	Webpage
	Texas	187	328	1703	5	
	Washington	230	446	1703	5	
	Wisconsin	265	530	1703	5	

**Table 4:** Accuracy (F1-score) of role inference across datasets and methods.

Methods/Acc	Enron	Cora	Citeseer	WEBkb				Mean Acc
				Cornell	Texas	Washington	Wisconsin	
Ember	0.65	-	-	-	-	-	-	0.65
Struct2vec	0.58	0.3	0.31	0.41	0.6	0.52	0.5	0.46
GCN	0.71	0.85	**0.68**	0.7	0.9	0.91	0.72	0.78
GAT	0.67	0.78	0.51	0.81	0.87	0.71	0.77	0.73
GWNN	0.66	0.82	**0.68**	0.76	0.65	0.9	0.74	0.74
SDNE	0.46	0.31	0.21	0.47	0.55	0.46	0.47	0.42
GraphSAGE-Mean	0.68	0.83	0.63	0.76	**0.94**	**0.94**	0.82	0.80
**Our Method**	**0.77**	**0.87**	0.6	**0.9**	0.88	0.85	**0.89**	**0.82**

**Table 5:** Accuracy of different aggregation strategies on baseline datasets.

Methods	Enron	Cora	Citeseer	WEBkb			
				Cornell	Texas	Washington	Wisconsin
Our Method	0.77	0.87	0.6	0.9	0.88	0.85	0.89
Our Method-Mean	0.72	0.57	0.36	0.79	0.74	0.73	0.82
Our Method-Att	0.61	0.82	-	0.83	0.83	0.89	0.79
GraphSAGE-Mean	0.68	0.83	0.63	0.76	0.94	0.94	0.82

**Table 6:** Time consumed by each layer.

Layer/Time (s)	Enron	Cora	Citeseer	WEBkb
Meanlayer	0.03	0.6	1.2	0.16
Attentionlayer	0.11	0.21	0.4	0.07
GateLayer	0.01	0.06	0.1	0.03
Sum	0.15	0.87	1.7	0.26

## Data Availability

The Enron email dataset is publicly available at https://www.cs.cmu.edu/~enron/, accessed on 28 October 2021. The Cora dataset is publicly available at https://relational.fit.cvut.cz/dataset/CORA, accessed on 28 October 2021. The Citeseer dataset is publicly available at https://networkrepository.com/citeseer.php, accessed on 28 October 2021. The WEBkb dataset is publicly available at http://www.cs.cmu.edu/~webkb/, accessed on 28 October 2021.
